# Microstructure Control of Welded Joints of Dissimilar Titanium Alloys by Isothermal Forging

**DOI:** 10.3390/ma13153347

**Published:** 2020-07-28

**Authors:** Yongqiang Zhang, Xiangyi Xue, Jingli Zhang, Huiming Li, Ping Guo, Hao Pan, Hongmiao Hou, Guoyu Jia

**Affiliations:** 1 State Key Laboratory of Solidification Processing, Northwestern Polytechnical University, Xi’an 710072, China; zhyq613@mail.nwpu.edu.cn (Y.Z.); gdz_zjl@163.com (X.X.); 2 Northwest Institute for Non-ferrous Metal Research, 96, Weiyang Road, Xi’an 710016, China; yf_zjl@163.com (P.G.); ph_zjl@163.com (H.P.); zsz_zjl@163.com (H.H.); zb_zjl@163.com (G.J.); 3 School of Materials Science and Engineering, Northeastern University, NO. 3-11, Wenhua Road, Heping District, Shenyang 110819, China; lyy_zjl@163.com

**Keywords:** dissimilar titanium alloys, electron beam welding, welded joint, isothermal forging, strengthen, dynamic globularization

## Abstract

In this study, the welded joints of dissimilar titanium alloys Ti600/Ti-22Al-25Nb were strengthened by isothermal forging. Different deformation parameters, including temperature, deformation speed, and reduction, were chosen. By isothermal forging, the original coarse dendritic grains of the welded joints were broken up effectively to form a large number of equiaxed grains. Meanwhile, many second phases were precipitated in the grain. Additionally, the dynamic globularization kinetics of second phases within the welded joints were quantitatively characterized and investigated. The results showed that the dynamic globularization kinetics and globularization rate were sensitive to the deformation conditions, and were promoted by a reduced strain rate and an elevated deformation temperature.

## 1. Introduction

Some key components of aero-engines, produced from a single titanium alloy, have routinely been used in complex environments for a long time. However, sometimes components composed of a single titanium alloy cannot meet the high performance requirements of aero-engines [1,2,3]. Therefore, it is necessary to explore the manufacturing technology of dual-titanium-alloy components. In recent years, lots of researchers have focused on dual-titanium-alloy welding, such as TC11/TC17 [4], TC4/TA7 [5], Ti22Al25Nb/TC4 [6], and so on [7,8,9,10,11]. However, due to the differences in physical properties and chemical composition between titanium alloys, the microstructure and properties of dual-titanium-alloy welded joints were always poor.

Post-weld heat treatment has been commonly used to improve the welded joint [12,13,14]. By this method, the microstructure to some extent can be controlled; however, the original large grains still cannot be broken. Thus, the welded joint sometimes becomes one of the weak links in the structural reliability of the product [15]. Recently, researchers have attempted to improve the welded joints by deformation. Isothermal forging was adopted by GE Aircraft Engines to strengthen the KM4/SR3 welded joint [16]. Tensile strengths in excess of 1378 MPa were achieved at 649 °C, with creep capability demonstrated at up to 760 °C. Mechanical testing across the KM4/SR3 joint resulted in failures in the base metal with strengths/lives equivalent to the base metal’s properties, confirming joint integrity. Osamu Tsuda et al. [17] effectively enhanced the bonding interface of the AF115/TMP-3 dissimilar alloy by superplastic isothermal forging, where the interface microstructure became fine and uniform. Lu et al. [18] used hot roller compaction to improve the metallographic structure and mechanical properties of the welded joints of coiled tubing. They found that the original coarse grains were significantly refined. Thus, the welded joint can be strengthened effectively by thermal deformation.

In this study, the high-temperature titanium alloy Ti600 and the Ti-22Al-25Nb alloy were welded by a vacuum electron beam welder, and then forged isothermally under different deformation parameters. The microstructure and properties of welding and forging were compared. The distribution of elements was investigated. The second phase precipitated in welded joints was analyzed and then the globularization model was established.

## 2. Materials and Methods

**Figure 1:**
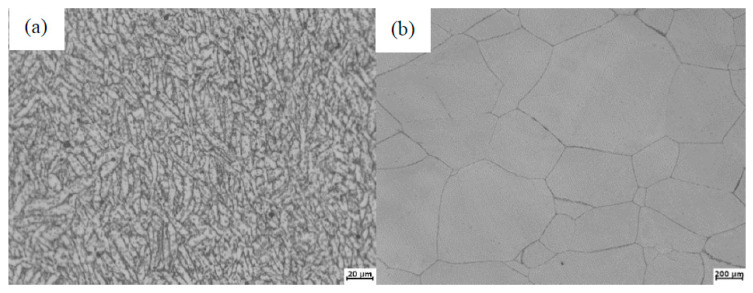
Microstructure of (**a**) Ti600 and (**b**) Ti-22Al-25Nb.

Ti600 is a kind of near-α high-temperature titanium alloy, developed by the Northwest Institute for Nonferrous Metal Research in China [19], whose chemical composition is Ti-6Al-2.8Sn-4.0Zr-0.5Mo-0.4Si-0.1Y (wt.%). With the addition of the rare earth element Y, Ti600 has good high-temperature creep performance and strength, and has been applied to some aerospace components. The other base metal used in this study is Ti-22Al-25Nb (at %, i.e., Ti-10.88Al-46.53Nb wt.%), a kind of Ti2AlNb alloy, with excellent creep and oxidation resistance [20]. From Figure 1, we found that the original microstructure of Ti600 is characterized by α lamellae, uniformly distributing in the β matrix, and that there are a lot of larger B2 grains within Ti-22Al-25Nb but that no second phase can be found.

**Figure 2:**
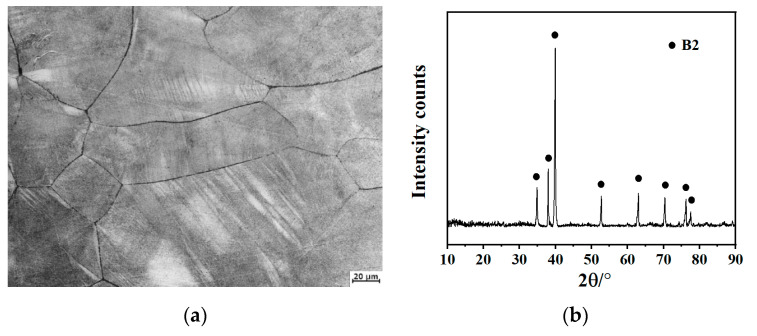
(**a**) Microstructure and (**b**) XRD of the Ti600/Ti-22Al-25Nb welded joint.

Both base metals used were in sheets of 20 mm thickness. Before welding, the welding surface was polished and cleaned with acetone to remove oil, water, and oxide. Double-sided welding was adopted to ensure complete penetration. The welding parameters are shown in Table 1. Firstly, the specimens were fixed in their positions by a weld with a small welding current, then welded on both sides, and finally defects on the weld surface were modified. After welding, the specimens were cooled slowly in a vacuum chamber for 10 min and then taken out. The complete welded joint was cut out by a wire cutting machine, and then polished and etched with the proper solution (HF: HNO_3_:H_2_O_2_:H_2_O = 1:2:7:20). The microstructures in this paper were investigated by ZEISS optical microscopy (OM) or HITACHI scanning electron microscopy (SEM, Tokyo, Japan), and phase composition was analyzed by a BRUKER X ray diffractometer (XRD, Billerica, MA, USA). Figure 2a shows the microstructure and XRD results of the welded joint. Note that the welded joint is composed of coarse grains, with no precipitates inside. The average grain size of the welded joints is about 225 μm. This result is in agreement with the XRD result (Figure 2b) that only B2 matrix existed in the welded joint.

**Figure 3:**
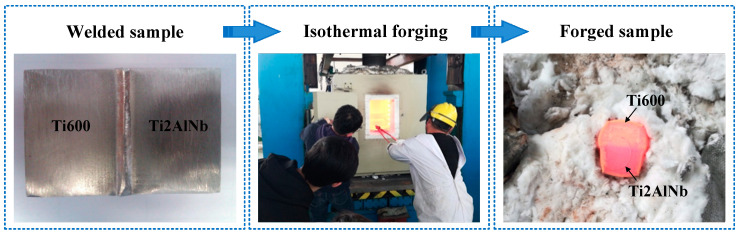
Flow diagram of isothermal forging.

Figure 3 is the flow diagram of isothermal forging. The specimen was compressed by a 600 t hydraulic press, and a heat holding furnace was installed outside the dies. When the dies were heated to the given temperature, the sample was put into the furnace for 20 min and then pressed. The experiment was conducted at 950–1050 °C and 0.005–0.1 mm/s, and different reductions (30%, 40%, and 50%) were also considered. After deformation, the samples were cooled in air. It was found that the base metal on both sides showed different resistances to deformation.

## 3. Results and Discussion

### 3.1. Microstructure Analysis of Welded Joints

**Figure 4:**
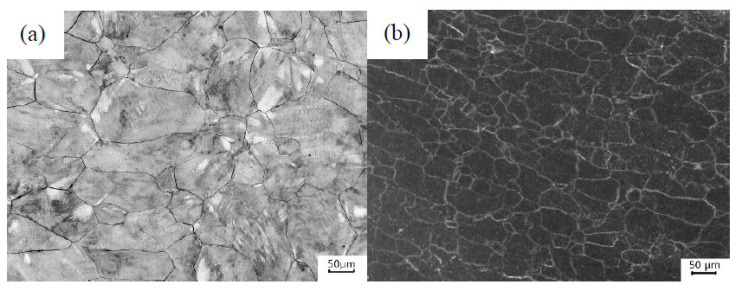
(**a**) The welded and (**b**) forged welded joints of Ti600/Ti-22Al-25Nb.

During isothermal forging, dynamic recrystallization takes place within the welded joint, under the action of thermal-mechanical coupling (Figure 4). The original coarse dendritic grains were broken up to form a large number of equiaxed grains. The dynamic recrystallization process occurred and the average grain size in the welded joint dropped below 60 μm. Meanwhile, many second phases were precipitated in the grain.

**Figure 5:**
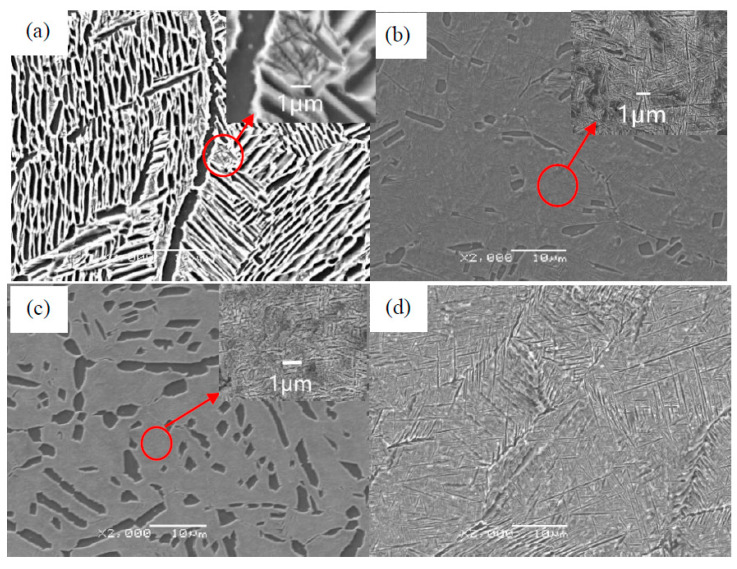
Microstructures of Ti600/Ti-22Al-25Nb welded joints after deformation at: (**a**) 950 °C; (**b**) 975 °C; (**c**) 990 °C; (**d**) 1010 °C.

From Figure 5, note that after isothermal forging the welded joint is composed of the matrix B2 phase, the O phase, and α_2_ phase [21]. The α_2_ phase is the darkest, and is lath-shaped or globular, and the matrix B2 phase is the lightest. The O phase is of medium color and has two forms. During forging, the O phase is formed around the α_2_ phase in a brim-shape. When cooled in air after forging, spicular O phase is precipitated in the matrix. From Table 2, the EDS result shows that the content of Al in the α_2_ phase is the highest, up to 12%, and the Nb content in the O phase is much higher than that in the B2 and α_2_ phases, which is 36%. Compared with the Ti-22Al-25Nb alloy, the B2 and α_2_ phases contain a small amount of Sn due to the mixing of Ti600 alloy.

As can be seen from Figure 5, when the deformation temperature ranges from 950 °C to 990 °C, the α_2_/O phase in the welded joint gradually becomes coarse. At the same time, some lath α_2_/O phase underwent spheroidization as the temperature elevated, and then formed spherical or necklace-shaped phase. When the deformation temperature was above 1010 °C, only spicular O phase could be observed in the B2 matrix. Therefore, it can be deduced that 1010 °C is higher than the phase transition point of the welded joint, and the second phase α_2_/O is completely transformed into B2 phase under these conditions.

Table 3 shows the tensile properties of welded and forged samples. Rm, Rp0.2, A, and Z are tensile strength, 0.2% yield strength, elongation, and reduction of area, respectively. The welded tensile sample was brittle, and broke at the welded seam without appreciable macroscopic deformation, and also exhibited low strength. By isothermal forging, the coarse grains of the welded joints were broken, and the matrix was strengthened by the secondary phase precipitates. Therefore, the mechanical properties of the forged sample were improved effectively. The forged samples broke on the Ti600 side, indicating that the strength of the welded joint was higher than that of Ti600. The fracture displayed a cup-and-cone type, which accounts for the ductile fracture.

### 3.2. Characterization of Dynamic Globularization Kinetics

According to the above analysis, a large amount of brittle second phase will be precipitated within Ti-22Al-25Nb/Ti600 welded joints at 950–990 °C, and globularized with the deformation. For most metals, the mechanical properties are always affected directly by the microstructure. Therefore, it is necessary to analyze the effect of thermal deformation parameters on the microstructure of welded joints, in order to provide a theoretical basis for mechanical properties’ improvement. In this study, in order to quantitatively study the dynamic globularization kinetics of the second phase within Ti-22Al-25Nb /Ti600 welded joints, thermal compression experiments on the welded joints were carried out at different temperatures (950 °C, 975 °C, and 990 °C), deformation strain rates (0.005 mm/s, 0.01 mm/s, and 0.1mm/s), and amounts (20%, 40%, 60%, and 70%).

**Figure 6:**
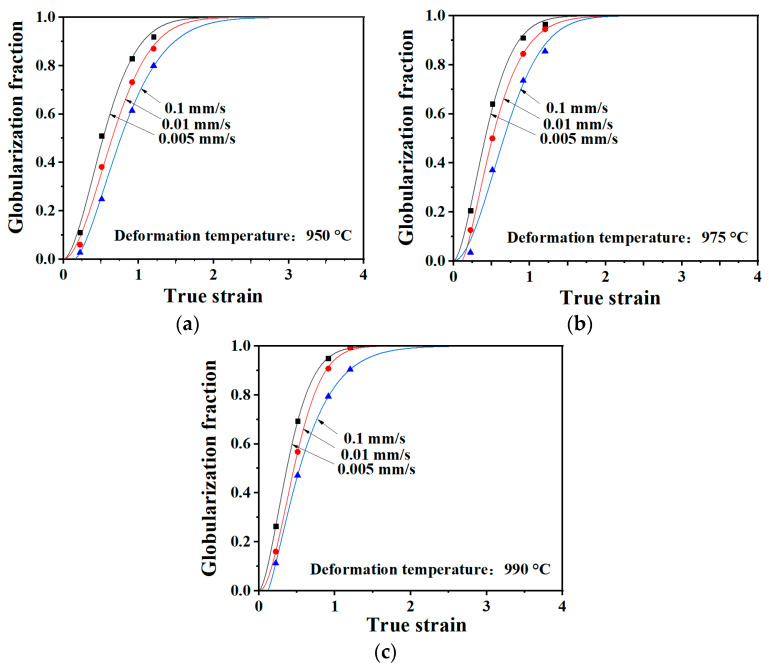
Theoretical dynamic globularization curves under: (**a**) 950 °C; (**b**) 975 °C; and (**c**) 990 °C.

The data points in Figure 6 are the measured globularization volume fractions of the second phase Ti-22Al-25Nb /Ti600 welded joints. It can be found that when the dynamic globularization process begins, the dynamic globularization degree of the second phase is very sensitive to both deformation temperature and velocity. The researchers found that the dynamic globularization of the second phase in titanium alloys can be described by the Avrami type equation, which has been applied to Ti-22Al-25Nb [22], TA15 [23], TC4 [24], and so on. The Avrami type equation can be expressed as

(1) $$f_{Dg}=1-\exp\left[-k{\left(\varepsilon -\varepsilon_{c}\right)}^{n}\right]$$

where *f_Dg_* is the globularization volume fraction of second phase; *ε* and *ε_c_* are the true strain and the critical strain of dynamic globularization, respectively; *n* is the Avrami exponent; and k is the kinetics constant related to temperature. In this equation, the parameters *ε_c_*, *n*, and *k* were all obtained by fitting.

The curves in Figure 6 are the theoretical dynamic globularization curves obtained by Equation (1), and are consistent with the measured data. Therefore, it may be reasonable to describe the dynamic globularization kinetics of Ti-22Al-25Nb/Ti600 welded joints by the Avrami type equation. The kinetics constant k varies between 1.42 and 3.51, and the Avrami exponent n ranges from 1.29 to 1.89. The theoretical dynamic globularization curves are s-shaped. There is a short gestation period of the curves in the initial stage, then a rapid increase after reaching the critical strain, and eventually the globularization slows down and the curves flatten out. In addition, dynamic globularization occurs earlier when the temperature elevates. From the shape of the curve, the theoretical globularization curves are similar to the dynamic recrystallization curves of many titanium alloys [25,26,27]. However, the above two processes are completely different. Dynamic recrystallization is essentially the migration of grain boundaries, including nucleation and growth of new grains, whereas new dynamic recrystallization grains cannot be observed during the globularization process. The brittle lamellae phase in the welded joint is partly broken up under external forces, and partly by forming grooves in the middle of the lamellar phase, which may be due to the formation of low- and high-angle α/β boundaries, or shear bands, across the α lamellae, followed by penetration of β phase to complete the separation.

In order to further analyze the dynamic globularization mechanism, the dynamic globularization rate $\nu_{\mathrm{Dg}}$ was introduced to characterize the globularization process, which can be given as [22,23]

(2) $$\nu_{\mathrm{Dg}}=\frac{\partial f_{Dg}}{\partial \varepsilon }$$

**Figure 7:**
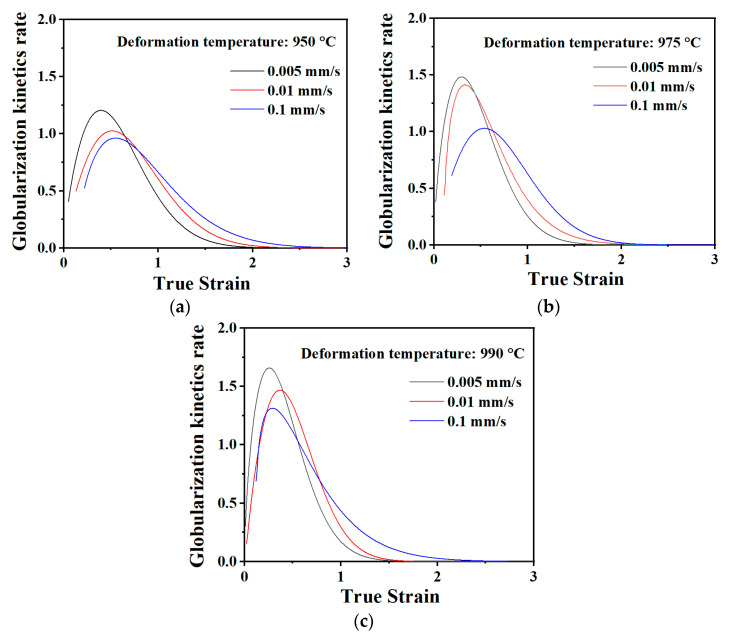
Globularization kinetics rate versus true strain curves under: (**a**) 950 °C; (**b**) 975 °C; and (**c**) 990 °C.

Figure 7 shows the globularization kinetics rate versus true strain curves under different conditions. As can be seen from the figure, when the strain reaches 0.2–0.7, the globularization kinetics rate curve of the second phase in the welded joints first increases to a peak sharply, mainly due to the fragmentation of the original lamellar phase. Then, as the aspect ratio of the lamellar phase decreases after fragmentation, continuous fragmentation of lamellar becomes more difficult, leading to a reducing globularization kinetics rate curve. Globularization is a diffusion-controlled process, which is affected by the deformation temperature and strain rate. Under the conditions of high temperature and a slow strain rate, driven by the release of distortion energy, the second phases are separated by the penetration of B2 phase at the defect position of the phase boundary.

## 4. Conclusions

(1) The welded joint of Ti600/Ti-22Al-25Nb contained coarse B2 grains without any precipitated phase. By isothermal forging, the original coarse dendritic grains were broken up effectively to form a large number of equiaxed grains. Meanwhile, many second phases were precipitated in the grain.
(2) When the deformation temperature was below 1010 °C, some lath α2/O phase within the welded joints underwent globularization, and then formed spherical or necklace-shaped phase. When the deformation temperature was above 1010 °C, only spicular O phase could be observed in the B2 matrix.
(3) The dynamic globularization kinetics of second phases were analyzed, and found to be in accordance with the Avrami type equation. The dynamic globularization kinetics and globularization rate were sensitive to deformation conditions. It was found that the process of dynamic globularization was promoted by decreasing the strain rate and increasing the deformation temperature.

## Figures and Tables

**Table 1:** Electron beam welding parameters of Ti600/Ti-22Al-25Nb specimens.

Welding Method	Accelerate Voltage /kV	Focusing Current /mA	Welding Current /mA	Welding Speed /mm·s^−1^
Position welding	150	1950	6	8
Welding	150	1950	22	8
Modify welding	150	1950	10	8

**Table 2:** Elementary composition of B2, α_2_, and O phases.

	Element	Ti	Al	Nb	Sn	Si
Phase						
B2		63.74	7.32	27.66	1.17	0.11
α_2_		68.43	11.79	17.16	2.14	0.49
O		59.48	7.33	36.20	2.31	-

**Table 3:** Tensile properties of the welded and forged samples.

	Properties	Rm/MPa	Rp0.2/MPa	A%	Z%
Samples					
**Welded sample**		713	686	-	-
Forged sample *		963	861	7.0	21.0

* Deformation conditions: 1010 °C, 40%, 0.01 mm/s.
